# Recovery of Hydroxytyrosol from Olive Pomace: Extraction, Purification, Bioactivity, and Bioavailability

**DOI:** 10.3390/foods15152672

**Published:** 2026-07-29

**Authors:** Jianing He, Tingting Li, Guanghui Hai, Caihong Zhang

**Affiliations:** 1 State Key Laboratory for Development and Utilization of Forest Food Resources, Nanjing Forestry University, Nanjing 210037, China; jianinghe068@njfu.edu.cn; 2 College of Light Industry and Food Engineering, Nanjing Forestry University, Nanjing 210037, China; 3 Longnan Academy of Non-Wood Forest, Longnan 746000, China; higuanghi@126.com; 4 State Key Laboratory for Development and Utilization of Forest Food Resources, Institute of Chemical Industry of Forest Products, Chinese Academy of Forestry, Nanjing 210042, China; zch@caf.ac.cn

**Keywords:** olive pomace, hydroxytyrosol, by-product valorization, extraction and purification, biological activities

## Abstract

Olive pomace (OP), the main by-product of olive oil production, is a rich source of phenolic compounds, among which hydroxytyrosol (HT) has received considerable attention because of its biological activity. This review examines the occurrence of HT in OP and the factors that influence its formation during processing, with particular attention to the intrinsic compositional variability arising from cultivar differences, irrigation regimes, fruit maturity, and extraction systems, which collectively challenge industrial reproducibility. Current strategies for HT extraction and purification are reviewed, and the technological readiness of each method is evaluated. The biological activities of HT, particularly its antioxidant, anti-inflammatory, and cardioprotective effects, are summarized alongside a critical assessment of in vivo bioavailability data. Key research gaps are identified throughout: no pilot-scale integrated purification train has been validated, life cycle assessments comparing different recovery routes are absent, and most delivery systems lack in vivo pharmacokinetic evaluation. By bringing together studies on HT recovery and its functional properties, this review highlights the promising yet underdeveloped potential of OP as a sustainable feedstock for HT recovery and identifies the critical steps needed to advance from laboratory research to industrial application.

## 1. Introduction

**Figure 1:**
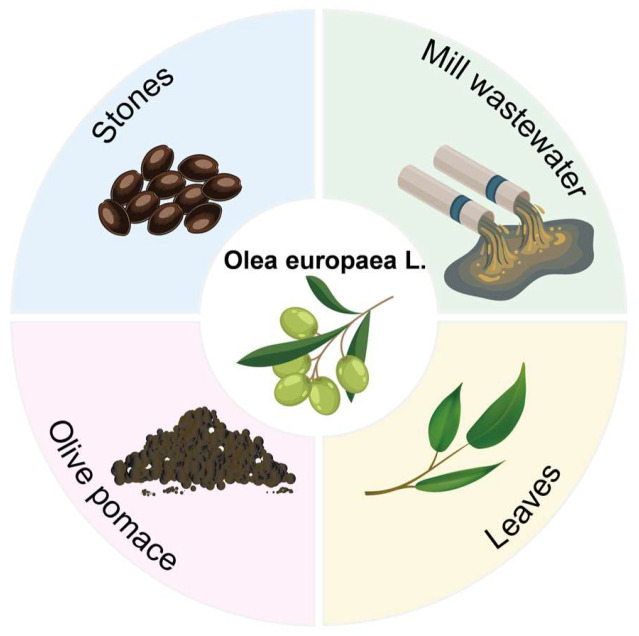
Four by-products generated during olive oil processing.

The olive tree (*Olea europaea* L.), an oil-bearing crop belonging to the genus Olea of the Oleaceae family, is suited to regions with mild, wet winters and hot, dry summers [1]. According to the International Olive Council [2], the global olive cultivation area for the 2024/2025 season was estimated at approximately 11.7 million hectares, with Spain accounting for the largest share at 2.7 million hectares. Driven by the continuous expansion of global cultivation areas, olive oil production has steadily increased, reaching approximately 2 million tons annually. Notably, Mediterranean countries account for 97% of this global production. Although this large-scale production yields significant economic and health benefits, it paradoxically imposes a severe environmental burden due to the generation of massive quantities of processing by-products (Figure 1), including pomace, mill wastewater, leaves, stones, and seeds [3]. Among these, olive pomace (OP) constitutes the predominant processing by-product, primarily consisting of pulp, skin, and stones. For every 100 kg of olives processed, approximately 70 to 80 kg of pomace is generated [4]. Furthermore, due to the seasonal nature of olive harvesting, enormous volumes of these by-products accumulate over a very short period, presenting substantial challenges to the sustainable development of the industrial chain.

Despite being a waste material, OP possesses a rich chemical composition, encompassing phenolics, lignans, flavonoids, dietary fiber, and proteins [5,6]. Its principal antioxidant compounds include phenolic compounds such as hydroxytyrosol (HT), tyrosol, and vanillic acid [7,8]. Among these, HT stands out as one of the most abundant polyphenols. It exhibits multifaceted physiological activities and confers substantial benefits to human health [9].

Consequently, maximizing the utilization of OP requires the efficient extraction and refinement of its high-value bioactive compounds. However, current research on these compounds is constrained by several bottlenecks, such as low extraction efficiency, complex purification processes, and fragmented mechanistic studies. As a result, most OP is discarded or merely used as animal feed [10], without being utilized effectively. Effective utilization of these processing by-products would not only alleviate environmental burdens but also maximize the recovery of valuable resources from olives. Therefore, this review systematically summarizes the natural sources, extraction, purification, and analytical detection technologies of HT derived from olive pomace, along with its biological activities. Furthermore, it discusses current challenges and future perspectives, with the overall aim of providing a robust theoretical foundation for the sustainable, high-value utilization of OP.

Several reviews have addressed HT recovery from olive by-products or its biological activities individually. However, a comprehensive review that integrates extraction, purification, bioactivity, and bioavailability with a critical assessment of technological maturity (TRL), scalability, and sustainability specifically for OP is currently lacking. This review fills this gap by providing a transversal analysis that connects biomass variability, extraction efficiency, downstream purification feasibility, and the impact of processing choices on the final bioactivity and bioavailability of HT. Furthermore, we discuss the industrial translation prospects and highlight the need for life cycle assessment (LCA) studies in this field.

## 2. Chemical Structure and Physicochemical Properties of HT

HT is a naturally occurring phenolic compound abundant in olives and their processing by-products (e.g., OP), typically presenting as a white to off-white crystalline powder. Chemically identified as 3,4-dihydroxyphenylethanol, it has the molecular formula C_8_H_10_O_3_ and a relative molecular mass of 154.16. Its core chemical structure features a benzene ring substituted with two adjacent phenolic hydroxyl groups at the 3- and 4-positions (constituting a catechol moiety), along with a hydroxyethyl side chain. This catechol moiety is the primary molecular determinant of its broad spectrum of biological activities [11,12].

The diverse applications of HT across the food, pharmaceutical, and agricultural sectors are fundamentally governed by its specific physicochemical properties. As a highly polar and hydrophilic molecule, it is readily soluble in polar solvents such as water, methanol, and ethanol. While relatively stable under acidic conditions, its stability is significantly compromised when exposed to alkaline environments, elevated temperatures, light, oxygen, and metallic ions. In the gas phase, the O-H bond dissociation enthalpy (BDE) of HT is approximately 333.7 ± 5.6 kJ·mol^−1^, which is substantially lower than that of its monophenolic analog, tyrosol (368.1 ± 5.6 kJ·mol^−1^). From a thermodynamic perspective, this lower BDE strongly explains its superior capacity to donate hydrogen atoms for free radical scavenging [13], thereby reinforcing its role as a highly potent antioxidant.

However, owing to the high reactivity of its hydroxyl groups, HT is extremely sensitive to ambient air and photodegradation, making it highly susceptible to degradation during food processing operations [14,15]. To mitigate these structural vulnerabilities and expand its applications, recent studies have focused on synthesizing HT derivatives. For instance, biotransformation and chemical modifications introducing lipophilic chains have emerged as effective strategies to significantly bolster its oxidative stability and overall antioxidant capacity in complex matrices [16].

## 3. Forms of HT in Olive Pomace and Influencing Factors

### 3.1. Forms of HT in Olive Pomace

HT in OP exists simultaneously in free, glycoside-bound, and esterified forms [17]. In fresh olives and their processing by-products, HT occurs predominantly in bound forms, primarily as oleuropein and other secoiridoid precursors [9]. Its actual content and structural distribution are significantly influenced by the olive variety, maturity, processing methods (e.g., crushing and extraction techniques), and storage conditions [18].

**Figure 2:**
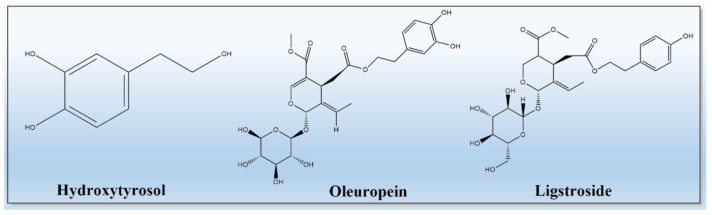
Chemical structures of representative phenolic compounds from olive (*Olea europaea*).

The generation of free HT relies primarily on the chemical and enzymatic degradation of these specific precursors (Figure 2). During post-harvest crushing and extraction, the cellular structure of the olive fruit is disrupted, allowing previously compartmentalized phenolic substrates to interact with endogenous enzymes such as β-glucosidases and esterases. This interaction triggers the cleavage of glycosidic and ester bonds, leading to the gradual release of free HT. Simultaneously, microbial metabolism and environmental factors during processing and storage can also facilitate the conversion of associated precursors into HT. Therefore, the HT content in pomace is essentially the synergistic result of precursor release, enzymatic hydrolysis, and subsequent biotransformations [19]. Microbial metabolism and environmental exposure further drive these biotransformations during subsequent storage.

Overall, the structural distribution of HT in OP is dynamic, with a significant fraction remaining bound as precursors within the matrix. Because natural degradation pathways seldom maximize the release of free HT, targeted processing interventions are critical to enhance its recovery. Overcoming this limitation requires a systematic approach. It is first necessary to understand the intrinsic variability of the starting biomass prior to applying any targeted post-harvest processing interventions.

### 3.2. Intrinsic Variability and Its Impact on Industrial Reproducibility

A major bottleneck in the industrial valorization of OP is its intrinsic compositional variability. OP is not a standardized feedstock; its physical and chemical properties fluctuate dramatically. These variations directly influence both the initial concentration of HT precursors and the final extraction efficiency, challenging industrial reproducibility.

Agronomic and environmental factors: Water availability during fruit development strongly influences the phenolic profile of olive fruits. Servili et al. [20] reported that deficit irrigation (DI) and severe deficit irrigation (SI) in cv. Leccino significantly increased total phenols and o-diphenols in virgin olive oil compared with full irrigation (FI) over two growing seasons. This effect extended to key secoiridoid derivatives: 3,4-DHPEA-EDA, 3,4-DHPEA-EA, and p-HPEA-EDA were all higher under water deficit than under full irrigation. García et al. [21] observed the same trend in cv. Arbequina. Total phenols, orthodiphenols, secoiridoid derivatives, and individual compounds such as HT decreased as the irrigation dose increased in both years of their study. These findings indicate that regulated deficit irrigation can enhance the phenolic content of olive fruits and, consequently, of the resulting pomace, although the magnitude of this effect varies with cultivar, growing season, and the severity of water stress.

Harvest conditions and maturity index: The ripening stage of olive fruits at harvest directly affects the phenolic composition of the resulting pomace. Amiot et al. [22] monitored the main phenolic compounds in three olive varieties during fruit development and maturation. Oleuropein, the principal HT precursor, reached up to 14% of the dry matter in young fruit but progressively declined as ripening advanced. During maturation, oleuropein undergoes enzymatic and chemical hydrolysis, gradually releasing free HT and elenolic acid [22]. Consequently, pomace from early-harvested green olives contains higher proportions of oleuropein and other bound forms of HT, whereas pomace from fully ripened black olives is richer in free HT. This compositional shift has practical implications. When the target is free HT, fully ripened fruit or controlled post-harvest storage of the pomace may be advantageous. Fernández-Prior et al. [23] tracked the evolution of HT and related compounds in alperujo over the harvest season. Free HT concentrations increased during the first three months of storage, fluctuated in the fourth month, and then stabilized. In contrast, HT 4-β-D-glucoside peaked at the beginning of the season and declined significantly by the second month [23]. These dynamics reflect the progressive enzymatic release of HT from its glycosidic precursors during pomace storage. For industrial HT recovery, the maturity stage of the original fruit and the storage history of the pomace must both be considered, as they jointly determine the quantity of HT available for extraction.

Extraction technology (two-phase vs. three-phase mills): Industrial origin is arguably the most critical determinant of pomace variability. The centrifugation process for olive oil extraction typically operates via either a three-phase or a two-phase system. Compared to the three-phase configuration, the two-phase system generates a lower volume of liquid olive mill wastewater (OMW) and a higher yield of wet pomace [9]. When ethyl acetate was employed to extract phenolic compounds from the residues of both systems, the total phenolic recovery from the two-phase system significantly surpassed that of the three-phase system. Subsequent analyses identified HT as one of the predominant phenolic compounds in these extracts. This highlights that the fundamental difference between the two milling technologies lies in water consumption and the consequent wastewater volume; because the three-phase system requires considerably more water, it results in greater phenolic leaching into the wastewater. Consequently, the two-phase system retains a significantly higher concentration of HT directly in the pomace [24].

### 3.3. Effect of Post-Harvest Processing and Pre-Treatments on HT Dynamics

**Figure 3:**
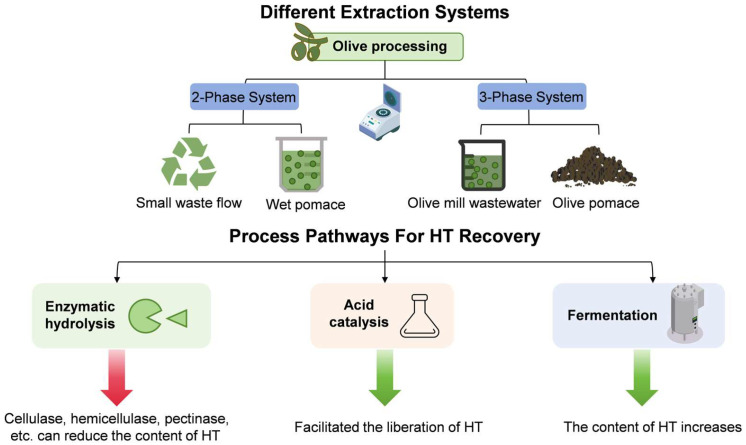
Different extraction systems and process pathways for hydroxytyrosol recovery.

As highlighted above, the physicochemical properties of OP are heavily dictated by the primary oil extraction system. However, prior to the final extraction phase, pomace is frequently subjected to various handling conditions or pre-processing pathways (Figure 3). It is crucial to recognize that these interventions act as a double-edged sword. Rather than unilaterally boosting HT yields, processes such as enzymatic hydrolysis, acid/base treatments, and fermentation profoundly alter the dynamic equilibrium of polyphenols. Depending on the processing parameters, these treatments can either facilitate the liberation of free HT from its precursors or, paradoxically, trigger its severe degradation.

Enzymatic hydrolysis: Research investigating the effects of various exogenous enzymes (cellulase, hemicellulase, pectinase, and neutral protease) and their dosages on HT extraction from OP revealed that the application of these enzymes significantly reduced the free HT content in the pomace. While varying the dosages of cellulase, hemicellulase, and pectinase measurably influenced the release dynamics of HT, variations in the concentration of neutral protease exhibited no significant impact [25].

Acid hydrolysis: In the hydrothermal treatment of olive wastes, comparative analyses between acid- and base-catalyzed treatments demonstrated that acid catalysis is clearly superior for HT extraction. Notably, under certain alkaline conditions, HT may undergo severe degradation due to base-catalyzed, free-radical-induced autoxidation, resulting in HT yields even lower than those obtained from uncatalyzed treatments [26].

Fermentation: Qualitative and quantitative analyses of OP subjected to both rapid and prolonged fermentation indicated significant structural shifts in protein, lipid, and phenolic compound profiles. Significantly, the HT content following prolonged fermentation was substantially higher than that observed following rapid fermentation, eventually determining HT as the most abundant phenolic compound in the fermented matrix [10].

In summary, the chemical state of HT in OP is inherently dynamic and highly susceptible to both natural and engineered factors. Intrinsic variables, such as cultivar, the specific maturity index, and primary milling technology, dictate the baseline phenolic profile of OP. Targeted post-harvest processing then serves as a critical lever to deliberately maximize the pool of free HT. However, industrial operators must maintain a delicate balance. Although enzymatic, acidic, and fermentative pre-treatments successfully liberate HT from complex secoiridoids, excessive exposure to prolonged oxidative environments or harsh conditions can trigger the degradation of newly released HT into quinones or inactive polymers. Therefore, it is the careful management of this pre-extraction phase that determines the maximum yield that can be achieved. The subsequent critical challenge lies in selecting an efficient and economically viable extraction technology to recover these target compounds from the complex matrix.

## 4. Extraction, Purification, and Determination of HT from Olive Pomace

### 4.1. Extraction of HT from Olive Pomace

OP is the most representative by-product of olive oil processing and is rich in phenolic bioactive compounds, particularly HT. Extraction methods differ markedly in operational complexity, extraction efficiency, and environmental impact. This section systematically describes several typical extraction techniques (Table 1).

#### 4.1.1. Solvent Extraction (SE)

Solvent extraction is a conventional technique for isolating HT from OP, predominantly utilizing aqueous, hydroethanolic, and organic solvent systems. Martins et al. [27] evaluated different green extraction methods and found that aqueous extraction at 50 °C exhibited excellent performance in total phenolic recovery and antioxidant capacity. However, it is not the most efficient method for HT extraction.

Similarly, Contreras et al. [28] compared two green extraction methods for recovering HT and mannitol from exhausted OP: hydrothermal extraction with pure water (AE-EOP) and ultrasound-assisted extraction with 40% acetone in water (AAE-EOP). The aqueous extract (AE-EOP) obtained at 85 °C for 90 min with 10% solid loading delivered a higher HT recovery yield (15.4 ± 0.5 mg/g extract) than the acetone-based ultrasound-assisted extract (8.7 ± 0.1 mg/g extract). AE-EOP also exhibited slightly stronger antioxidant activity. As a green chemistry approach utilizing pure water, AE-EOP features high safety and low equipment requirements. However, it is hampered by prolonged processing times (90 min), limited selectivity, and the risk of thermal degradation at the elevated operating temperature. In contrast, AAE-EOP required only 12 min of extraction time but used acetone, which is not food-grade and must be thoroughly removed from the final product.

#### 4.1.2. Ultrasound-Assisted Extraction (UAE)

Ultrasound-assisted extraction (UAE) leverages the acoustic cavitation effect to generate intense localized shear forces that compromise plant cell walls, thereby accelerating the mass transfer of HT and secoiridoids (e.g., oleuropein) into the solvent [29]. The composition of the extraction solvent plays a key role in UAE efficiency, as ethanol concentration influences solvent polarity and its interaction with HT [30].

Martins et al. [27] applied ultrasound-assisted hydroethanolic extraction (USAHE) to EOP using 90% ethanol at 50 °C for 120 min, with ultrasound applied via a probe (20 kHz, 10 min). This method achieved an HT content of 2.021 ± 0.29 mg/100 mg extract, the highest among all methods tested in their study, along with tyrosol and catechol. Madureira et al. [31] employed response surface methodology to optimize UAE parameters for HT and tyrosol recovery from OP. Under optimized conditions (490 W, 28 min, 7.3% ethanol), the HT content reached 36 ± 2 mg/g extract, with an extraction yield of 30 ± 2%. Compared with heat-assisted extraction (HAE), UAE reduced extraction time and solvent consumption while substantially increasing extraction yield. However, the UAE extract exhibited lower antioxidant, antidiabetic, anti-inflammatory, and antibacterial activities than the corresponding HAE extract, suggesting that extraction efficiency does not always correlate with bioactivity.

#### 4.1.3. Microwave-Assisted Extraction (MAE)

Microwave-assisted extraction (MAE) operates based on the continuous dipole rotation of polar molecules under a high-frequency electromagnetic field, which generates substantial internal heat and pressure, ultimately leading to cell wall rupture. Gómez-Cruz et al. [32] applied microwave-assisted water extraction (MAWE) to recover HT and mannitol from exhausted olive pomace (EOP). Using response surface methodology, the extraction parameters were optimized at 100 °C, 16 min, and 12% (*w*/*v*) solid loading. Under these conditions, a maximum HT content of 5.87 ± 0.28 mg/g EOP was achieved, with HT identified as the predominant phenolic compound in the extracts. Compared to conventional hydrothermal extraction of the same feedstock (85 °C, 90 min), MAWE achieved comparable phenolic recovery while reducing extraction time by 82% (16 vs. 90 min). Moreover, the use of water as the sole solvent aligns with green chemistry principles and facilitates subsequent downstream processing. Notably, the extracts also contained mannitol (46.70 mg/g EOP), demonstrating the potential of MAWE for the simultaneous recovery of multiple valuable compounds in a biorefinery framework.

#### 4.1.4. Enzyme-Assisted Extraction (EAE)

Enzymatic catalysis disrupts the structural matrix of OP, promoting the release of polyphenols. Martins et al. [27] showed that adding exogenous enzymes, such as cellulases and viscoenzymes, to hydroethanolic or aqueous extractions markedly improved recovery yields. The highest HT concentration (0.541 ± 0.031 mg/100 mg extract) was obtained with viscoenzyme-assisted aqueous extraction at 50 °C. Macedo et al. [33] evaluated the effect of cellulase, pectinase, and tannase on the aqueous extraction of phenolics from OP. The experiments were carried out at pH 5.0, 50 °C, for 120 min, with a solid-to-liquid ratio of 1:15 and 2.0% enzyme (*w*/*w*). Among the enzymes tested, the combination of pectinase and cellulase yielded the highest HT content (26.1 ± 0.1 mg/kg OP). These results confirm that enzyme-assisted extraction enhances HT recovery, although the long extraction time (120 min) remains a practical limitation.

#### 4.1.5. Supercritical Fluid Extraction (SFE)

Supercritical fluid extraction (SFE) capitalizes on the extraordinary diffusivity and tunable solvating power of fluids in their supercritical state. Caballero et al. [34] used supercritical CO_2_ enhanced with 60% ethanol as a co-solvent to extract polyphenols from olive wastes under different pressure conditions. Their data indicated that an operating pressure of 300 bar optimized the HT yield, reaching 1.25 mg/g olive waste extracts. Furthermore, subsequent studies on supercritical defatted OP [35] using pressurized liquid extraction (184.0 °C, 90.0% ethanol, liquid-to-solid ratio of 0.8 mL/g) achieved an impressive HT recovery of 9.5 mg/g dry extract. Undeniably, SFE offers excellent environmental safety and tunability. However, its widespread industrial implementation is currently limited by complex operating procedures and high equipment costs.

#### 4.1.6. Natural Deep Eutectic Solvents Extraction (NADES)

Conventional extraction methods using organic solvents have significant drawbacks, including toxicity, high volatility, and excessive solvent consumption. In contrast, deep eutectic solvents (DES), particularly natural deep eutectic solvents (NADES), are attracting increasing research attention. This is attributed to their environmentally friendly and sustainable properties [36].

In recent years, numerous studies have explored the use of NADES for extracting HT and other phenolic compounds from OP. Cabrera et al. [37] applied a lactic acid-glucose (La-Gc) NADES to recover antioxidant phenolic compounds from Uruguayan OP. The extraction process was optimized via RSM. The optimal conditions were 80 °C, a solid–liquid ratio of 0.014 g/mL, and a water content of 68% (*w*/*w*) in the La-Gc system. Under these conditions, the total phenolic content reached 15.56 mg GAE/g db, and the HT content was 1.24 mg/g ± 0.05 d.b.

NADES has also been combined with physical auxiliary techniques to further enhance extraction performance. Chanioti and Tzia [38] systematically compared several innovative methods (HAE, MAE, UAE, HHPAE) coupled with different NADES formulations. Among all tested combinations, homogenization-assisted extraction with a maltose-based DES (DES-MA) achieved the highest HT recovery of 6.08 ± 0.33 mg/g d.w. under conditions of 60 °C, 12,000 rpm, and 30 min. HPLC analysis further confirmed that NADES-based systems generally outperformed aqueous ethanol and pure water in extracting diverse phenolic compounds. These findings demonstrate that the integration of NADES with appropriate physical intensification techniques can remarkably enhance extraction efficiency.

The sustainability and biodegradability of NADES highlight their potential as green solvents for phenolic compound extraction from OP. Overall, NADES-based extraction represents a sustainable and efficient alternative to conventional organic solvents for recovering HT from OP. However, whether the extracts can be directly used or require further purification remains unclear.

**Table 1:** Comparison of extraction methods for hydroxytyrosol from olive pomace.

Method	Solvent System	HT Yield	Extraction Conditions	Main Advantages	Main Limitations	Ref.
SE	Water	15.4 ± 0.5 ^a^	85 °C, 90 min, S/L 1:10, water bath, 200 rpm	High safety; Low equipment requirements	Long time (90 min); Low selectivity; Thermal degradation risk	[28]
UAE	Ethanol: Water (9:1, *v*/*v*)	20.21 ± 2.87 ^a^	50 °C, 120 min, S/L 1:30, US probe 20 kHz (30 s pulses, 10 min)	High efficiency; Shorter reduction time vs. CSE; Broad applicability;	Lower bioactivity vs. HAE; No antifungal activity; Reduced cytotoxicity	[27]
	Ethanol: Water (7.3:92.7, *v*/*v*)	36 ± 2 ^a^	490 W, 28 min, RSM-optimized			[31]
MAE	Water	5.87 ± 0.28 ^b^	100 °C, 16 min, S/L 1:8.3, 850 W, 30 mL reactor	Shorter extraction time; lower energy consumption; reduced solvent use	High T (100 °C); MW penetration limits; equipment investment	[32]
EAE	Water (viscoenzyme-assisted)	0.00541 ± 0.0003 ^a^	50 °C, 120 min, S/L 1:30, 120 rpm, viscoenzyme	Enhanced HT recovery	Long extraction time; high cost	[27]
	Water (enzyme-assisted, pH 5.0)	0.0261 ± 0.0001 ^c^	50 °C, 120 min, S/L 1:15, 2% enzyme (*w*/*w*), 120 rpm			[33]
SFE	Supercritical CO_2_ + 60% (*v*/*v*) ethanol as co-solvent	1.25 ± 0.01 ^a^	300 bar, 50 °C, 60 min, S/L 1:3, 254 mL reactor	High environmental safety; tunable solvating power	Complex operation; high equipment cost	[34]
	Ethanol: Water (9:1, *v*/*v*)	9.5 ^a^	184 °C, 20 min, S/L 1:0.8, 10 MPa (N_2_), 750 rpm; Pre-treat: scCO_2_ 30 MPa, 60 °C, 3 h			[35]
NADES extraction	La-Gc NADES with 68% (*w*/*w*) water	1.24 ± 0.05 ^d^	80 °C, S/L 1:71, RSM-optimized	High extraction efficiency; green and sustainable	Need for further purification before direct application in food/cosmetic products remains unclear	[37]
	DES-MA	6.08 ± 0.33 ^e^	60 °C, 12,000 rpm, 30 min			[38]

Note: ^a^ mg/g extract (dry extract weight basis); ^b^ mg/g EOP (exhausted olive pomace, dry weight basis); ^c^ mg/g OP (olive pomace, dry weight basis); ^d^ mg/g d.w. (dry weight of starting material); ^e^ mg/g d.b. (dry basis). Values with superscript ^a^ are based on dry extract weight; values with superscripts ^b–e^ are based on dry weight of the starting olive pomace. Direct numerical comparison across different reference bases should be made with caution. Abbreviations: CSE, conventional solvent extraction; DES-MA, maltose-based deep eutectic solvent; EAE, enzyme-assisted extraction; HAE, heat-assisted extraction; HT, hydroxytyrosol; La-Gc, lactic acid-glucose; MAE, microwave-assisted extraction; MW, microwave; NADES, natural deep eutectic solvent; RSM, response surface methodology; SE, solvent extraction; SFE, supercritical fluid extraction; S/L, solid-to-liquid ratio; UAE, ultrasound-assisted extraction; *v*/*v*, volume per volume; *w*/*w*, weight per weight; Ref., Reference.

#### 4.1.7. Comparative Assessment and Industrial Perspective of Extraction Technologies

Selecting an extraction technology for industrial-scale HT recovery requires balancing yield, operational complexity, and cost. Conventional solvent extraction and UAE operate at high technology readiness levels (TRL 7–8) and scale up easily using existing processing infrastructure [28,31]. MAE (TRL 4–5) and EAE (TRL 5–6) have also shown promise, but they demand specialized equipment or expensive enzymatic reagents that slow their industrial adoption [27,32]. At the lower end, SFE (TRL 4–5) and NADES extraction (TRL 3–4) remain the furthest from commercial readiness [34,37]. This wide TRL gap means that near-term industrial implementation will likely rely on the more mature technologies, while emerging methods require further development before they can compete at scale.

A practical challenge that complicates technology selection is that higher yield does not always mean better product quality. UAE recovers large amounts of HT from OP, yet the resulting extracts show lower antioxidant and anti-inflammatory activities than those obtained by simple hot water extraction [31]. This might be because the intense acoustic energy damages sensitive bioactive structures or pulls out interfering compounds alongside HT. Hydrothermal extraction avoids organic solvents and achieves respectable yields, but the long heating time at high temperature creates a risk of thermal degradation [28]. MAE shortens this heating step considerably, yet it still needs careful temperature control to prevent localized overheating [32]. These trade-offs mean that an extraction method cannot be judged on yield alone; the functional quality of the final extract matters just as much.

Beyond the extraction step itself, what happens downstream after extraction also shapes the overall viability of a method. Aqueous and hydroethanolic extracts from SE, MAE, and EAE are straightforward to process. They can be fed directly into membrane filtration or adsorption columns without needing extra solvent removal steps [28,32,33]. SFE with carbon dioxide is attractive in this regard because the gas simply separates from the product when the pressure is released [34]. The picture changes when ethanol is used as a modifier, since a distillation step must then be added [35].

Perhaps the most troublesome case is NADES-based extraction. These eutectic solvents form strong hydrogen-bonded networks. They are viscous and practically non-volatile, which makes standard distillation or liquid–liquid extraction ineffective for pulling HT back out of the mixture [37,38]. Beyond the recovery challenge, the safety of NADES for food-grade applications has not been systematically evaluated. Despite their natural origin, these solvents lack regulatory approval as food-grade extraction media. Whether residual solvent components in the final extract pose any toxicological risk remains an open question. For industrial biorefineries, the combined difficulty of product recovery and the uncertain safety profile represent major barriers that must be weighed against the green solvent advantages claimed for these systems.

A comparison of these aspects is provided in Table 2.

### 4.2. Separation and Purification of HT from Olive Pomace

#### 4.2.1. Membrane Separation

Membrane technology serves as an efficient and straightforward strategy for the preliminary enrichment of HT. Nunes et al. [39] compared three polymeric membranes (NF270, NF90, and BW30) for treating aqueous extracts of OP. NF270 and NF90 are nanofiltration membranes with relatively loose rejection layers. HT, with a molecular mass of 154 g/mol, is small enough to pass through NF membranes. In their study, the NF270 permeate still contained a small amount of phenolics (4.4 mg GAE/L), whereas the BW30 reverse osmosis membrane achieved 100% rejection of phenolic compounds and flavonoids, with no detectable phenolics in the permeate and a total phenolic content of 1234.3 mg GAE/L in the concentrate. The BW30 membrane also exhibited the lowest fouling index and maintained a nearly constant permeate flux throughout operation. In contrast, NF270 and NF90 suffered from significant fouling.

Romeu et al. [40] exploited this principle of NF selectivity by developing a pilot-scale process combining nanofiltration (NF270 membrane) with reverse osmosis. The process was operated directly in an olive mill using standard industrial equipment. HT and tyrosol passed through the NF membrane into the permeate, while larger polyphenols were retained. Subsequent reverse osmosis (RO) concentration yielded a retentate with a 7- to 9-fold enrichment in HT relative to the feed. The HT content in the dry residue of the RO retentate reached 15%. The final product remained stable for over 14 months at room temperature. The operation processed 1500 kg of OP in one working week, producing approximately 80 L of concentrated aqueous solution containing up to 1.2 kg of HT.

Despite these promising results, membrane-based purification has inherent limitations. The HT concentration in the final RO retentate depends directly on the free HT content of the feed, which varies with OP storage time, cultivar, and harvest season [39]. Membrane fouling remains a practical concern, and regular cleaning is required to restore flux performance [39,40].

#### 4.2.2. Liquid–Liquid Extraction (LLE)

Liquid–liquid extraction remains one of the classical methodologies for the initial purification of HT. Gómez-Cruz et al. [41] systematically compared membrane separation, LLE, and solid-phase adsorption technologies for purifying HT from EOP aqueous extracts. Using ethyl acetate as the extraction solvent (1:2 *v*/*v* extract-to-solvent ratio, two sequential steps, 15 min stirring), LLE achieved the highest HT recovery of 88.8% among all tested methods. The resulting ethyl acetate fraction contained 118 mg HT per gram of extract and 273 mg total phenolics per gram of extract. This represented an 8-fold increase in HT purity compared with the original aqueous extract. Moreover, glucose and mannitol were almost completely retained in the aqueous phase, with less than 4% of these compounds partitioning into the organic phase. This high selectivity for HT over co-extracted components is an advantage for producing HT-enriched fractions without additional purification steps.

For analytical-scale applications, a more refined LLE-based approach has been developed. Habibi et al. [42] combined microwave-assisted extraction with dispersive liquid–liquid microextraction (MAE-DLLME) for the determination of oleuropein and HT in OP. The method used 1-octanol as the extraction solvent and ethanol as the dispersive solvent. Under RSM-optimized conditions (220 W microwave power, 12 min extraction time, 60 μL 1-octanol, 500 μL ethanol), recoveries of 98.7% for HT and 93.4% for oleuropein were achieved, with a detection limit of 20 μg/L for HT. However, this method was designed for analytical quantification rather than preparative purification, and 1-octanol is not a food-grade solvent.

#### 4.2.3. Solid-Phase Extraction Purification

Gómez-Cruz et al. [41] evaluated eight adsorbents and resins (C18, C8, HLB, Microionex MB 200, XAD7HP, XAD16N, Macronet MN202, and Purosorb PAD910) for the purification of HT from an aqueous extract of exhausted OP. Two elution methods were tested: Method 1 used methanol as the eluent, while Method 2 employed ethyl acetate followed by methanol. The performance depended on both the stationary phase and the eluent. Methanol generally provided higher recovery values for total phenolic compounds, whereas ethyl acetate was more selective for HT.

Among all tested combinations in the study, DSC-8 with ethyl acetate as the eluent yielded the highest HT purity of 291 mg/g extract and a TPC of 873 mg/g extract. The HT recovery in the ethyl acetate fraction was 30.85%, which accounted for virtually all of the total HT recovery (31.5%). The subsequent methanol elution contributed only 0.69% to HT recovery [41]. Importantly, both glucose and mannitol were almost completely retained in the aqueous phase during SPE, confirming the selectivity of this purification approach for phenolic compounds [41].

#### 4.2.4. Crystallization and Derivatization

HT in its unformulated state presents practical handling difficulties. At ambient temperature, it readily absorbs moisture and becomes a viscous liquid. It is also highly susceptible to oxidative degradation due to the catechol moiety in its structure. Cocrystallization has been explored as a strategy to overcome these limitations. Zhu et al. [43] prepared four cocrystals of HT with betaine (HT-BET), nicotinamide (HT-NIC), and isonicotinamide (HT-INA, Forms I and II). After cocrystallization, all products were solid powders with good flowability at room temperature. The melting points increased significantly compared with that of pure HT. HT-BET melted at 148.0 °C and HT-NIC at 109.6 °C, whereas pure HT melted at 53.2 °C. Dynamic vapor sorption tests showed that HT-NIC, HT-INA Form I, and HT-INA Form II absorbed less than 1% water at 95% relative humidity. Under accelerated chemical stability conditions (40 °C, 75% RH, sealed in double-layer polyethylene bags), HT-BET and HT-NIC showed no HT degradation after three months. Over the same period, pure HT retained up to 72.4% of its initial content, while encapsulated HT powder dropped to 34.0% and olive leaf extract to only 8.0%, and the two edible cocrystals showed the best stability without any degradation.

An alternative approach is chemical derivatization. Capasso et al. [44] converted HT into its triacetyl derivative using a mixture of perchloric acid adsorbed on silica gel (HClO_4_-SiO_2_) and acetic anhydride (Ac_2_O). After a single MPLC purification step, the triacetyl derivative was obtained with an overall yield of 35.6% from the organic extract of olive mill wastewater. The purified derivative showed no ferric reducing activity in chemical assays. However, in cellular tests using Caco-2 cells and human erythrocytes, it protected against oxidative stress to the same extent as free HT. This protective effect was attributed to enzymatic hydrolysis of the acetyl groups by cellular esterases, which regenerates the active HT. The triacetyl form offers practical advantages. It is chemically stable during storage and does not undergo oxidative degradation, unlike free HT. Despite these promising properties, the acetylated form has not been evaluated for therapeutic efficacy or safety at pharmacological doses. These aspects require further investigation before any clinical application can be considered.

The separation and purification methods for HT from OP are compared in detail in Table 3.

#### 4.2.5. Comparative Evaluation and Biorefinery Integration of Purification Schemes

The purification methods reviewed in the preceding sections differ substantially in their TRL and suitability for industrial deployment. Membrane separation and LLE are the most mature options. Both have been validated at the pilot scale, with membrane technology demonstrated directly in an olive mill setting [40] and LLE achieving the highest HT recovery among all tested methods [41]. These two techniques can be implemented using existing industrial infrastructure and are compatible with the aqueous or hydroalcoholic extraction systems described in Section 4.1. SPE, although operated at the laboratory scale, achieved the highest HT purity among the methods evaluated by Gómez-Cruz et al. (291 mg/g extract with DSC-8 adsorbent and ethyl acetate elution [41]), suggesting that further scale-up efforts are warranted. At the lowest readiness level, crystallization and derivatization remain confined to bench-scale studies. While both approaches effectively address the poor physicochemical stability of HT, neither has been evaluated for food safety or therapeutic use [43,44]. MAE-DLLME, owing to its design as an analytical method rather than a preparative purification technique, is not considered further in this section.

A clear trade-off emerges between recovery yield and final purity across these methods. LLE with ethyl acetate recovers nearly 90% of the HT from the feed but produces an extract of moderate purity (118 mg/g) [41]. SPE with DSC-8, by contrast, yields a product roughly three times as pure but recovers only about one-third of the initial HT [41]. This inverse relationship between purity and recovery is a common feature of separation processes and must be considered when selecting a purification strategy for a given application. For uses that tolerate lower purity, such as crude antioxidant additives, LLE may suffice. For pharmaceutical or high-purity nutraceutical applications, a combination of steps would be required, including membrane pre-concentration, SPE enrichment, and final purification by crystallization.

The feasibility of constructing such integrated purification schemes depends not only on the performance of each unit operation but also on their compatibility with each other and with the preceding extraction step. Aqueous extracts from solvent extraction, MAE, and EAE can be fed directly into a membrane filtration unit without solvent exchange or pH adjustment [39,40]. The retentate or permeate from membrane processing can then be directed to a resin adsorption column for further enrichment, following the approach demonstrated by Gómez-Cruz et al. [41]. In principle, after further enrichment, the concentrated extract from the SPE step could serve as feed for crystallization, yielding a solid product with excellent storage stability [43]. This cascading logic, in which a relatively crude extract is progressively refined through sequential purification steps, aligns with the biorefinery concept that has been proposed for OP valorization. Gómez-Cruz et al. illustrated how HT extraction and purification can be integrated with the recovery of triterpenic acids, antioxidant lignin, and fermentable sugars from the same starting material [41]. Including HT purification in such a framework would diversify the product portfolio and improve the overall economics of the biorefinery.

However, several key challenges must be addressed before these integrated schemes can be scaled up to industrial production. No study has yet connected membrane pre-concentration, resin-based enrichment, and crystallization into a single, sequential purification train, even at the laboratory scale. The performance of SPE resins over repeated adsorption–desorption cycles has not been systematically characterized, and the long-term stability of cocrystal and triacetyl products in real food or pharmaceutical matrices is unknown [43,44]. Solvent recovery, waste management, and the energy demand of each purification step must also be quantified through techno-economic analysis and life cycle assessment. These data are essential for evaluating the environmental sustainability of different purification routes beyond the qualitative comparisons presented in Table 4. Addressing these gaps will be critical for moving HT purification from the laboratory to industrial practice.

### 4.3. Detection and Quantification Methods for HT

#### 4.3.1. HPLC-DAD

For the precise characterization of HT and associated phenolic compounds in OP, high-performance liquid chromatography coupled with a diode-array detector (HPLC-DAD) represents the most widely used quantitative analytical method. Current literature reports an array of gradient elution methodologies predominantly relying on reversed-phase C18 analytical columns [27,35]. The binary solvent system is typically composed of an acidified aqueous phase (e.g., phosphoric acid to pH 3 [35] or trifluoroacetic acid [27]) and an organic modifier (e.g., methanol or acetonitrile) [27,35].

The selection of monitoring wavelengths is determined by the ultraviolet (UV) absorption characteristics of the target analytes. HT exhibits a characteristic UV absorption spectrum with a maximum absorption wavelength (λmax) at approximately 280 nm [45]. Tyrosol displays a comparable but slightly shifted absorption maximum at approximately 275.7 nm [27]. For quantitative analysis, the external standard method is commonly adopted, using pure HT standards to construct calibration curves; final results are generally normalized and expressed on a dry weight basis (e.g., mg/g or mg/100 mg of dry extract) [27,35].

#### 4.3.2. HPLC-MS/MS

While HPLC-DAD provides reliable quantitative data based on retention time and UV spectral matching, definitive structural confirmation of HT requires mass spectrometric detection. The application of HPLC-MS/MS for the detection of HT in OP enables the qualitative and quantitative analysis of various phenolic compounds in the matrix. Grigoletto et al. [8] coupled an HPLC system with a triple quadrupole (QqQ) mass spectrometer. Chromatographic separation was performed on a Poroshell 120 SB-C18 column (100 × 3.0 mm, 2.7 μm) at 35 °C, using gradient elution with mobile phase A [1% (*v*/*v*) acetic acid in water] and mobile phase B (acetonitrile) at a flow rate of 0.6 mL min^−1^. Detection was optimized in negative ESI mode using multiple reaction monitoring (MRM), and 42 phenolic compounds were tentatively identified [8].

In the negative electrospray ionization mode, HT yields a deprotonated molecular ion [M-H]^−^ at *m*/*z* 153 [8,35]. Tandem MS fragmentation produces a characteristic fragment ion at *m*/*z* 123 [8,35], consistent with the loss of 30 Da from the precursor ion. Grigoletto et al. [8] identified this as the best transition for MRM quantification, with a fragmentor voltage of 100 V and a collision energy of 10 eV. No other transitions for HT were reported in the optimized MRM method [8,35]. The complete set of optimized mass spectrometric parameters is summarized in Table 5.

## 5. Biological Activities of HT Derived from Olive Pomace

As a natural phenolic compound predominantly recovered from OP, HT has garnered increasing interest in recent years due to its diverse bioactivities. Current studies suggest that HT exerts important physiological effects across multiple domains, most notably through antioxidant, anti-inflammatory, cardiovascular-protective, anti-tumor, osteoprotective, and hypoglycemic activities. The current research progress is systematically summarized below.

### 5.1. Antioxidant Activity

HT is widely recognized as one of the most potent natural phenolic antioxidants. Its antioxidant activity is primarily attributed to two mechanisms: direct scavenging of free radicals accompanied by elevated intracellular glutathione (GSH) levels, and enhancement of antioxidant enzyme activities. In vitro assays have demonstrated that HT effectively scavenges test radicals such as DPPH and ABTS while also increasing intracellular GSH content in cell-based models [46], thereby protecting against oxidative stress-induced cellular damage [28]. In a CCl_4_-induced liver injury rat model, an OP extract rich in HT significantly upregulated the activities of catalase (CAT), superoxide dismutase (SOD), and glutathione peroxidase (GPx), facilitated the removal of free radicals generated during CCl_4_ metabolism, and inhibited lipid peroxidation, ultimately mitigating hepatic tissue damage caused by oxidative stress [28].

### 5.2. Anti-Inflammatory Activity

HT exerts anti-inflammatory effects by modulating a range of inflammation-associated signaling pathways and gene expression. In OP juice (OPJ), HT is the principal bioactive component. An investigation into the protective effects of OPJ against lipopolysaccharide (LPS)-induced inflammation in BV2 microglial cells showed that OPJ inhibited the NF-κB and MAPK pathways, leading to a significant downregulation of key inflammatory mediators, including inducible nitric oxide synthase (iNOS), cyclooxygenase-2 (COX-2), and the pro-inflammatory cytokines interleukin-6 (IL-6) and interleukin-1β (IL-1β), thereby suppressing neuroinflammation [47]. HT may also be effective against immune–inflammatory diseases such as systemic lupus erythematosus (SLE). Aparicio-Soto et al. [48], using an enzyme-linked immunosorbent assay (ELISA), found that dietary supplementation with HT and HT acetate in mice markedly inhibited three key inflammatory signaling cascades—JAK/STAT, MAPK, and NF-κB—resulting in a significant reduction in pro-inflammatory cytokines in peritoneal macrophages and splenocytes.

### 5.3. Cardiovascular-Protective Activity

HT plays a pivotal role in the prevention and management of cardiovascular diseases (CVDs). In an in vivo proteomic study employing nano-liquid chromatography coupled with mass spectrometry (nano-LC-MS), Catalán et al. [49] analyzed the aortic and cardiac tissues of healthy female rats supplemented with HT. Their findings revealed that HT actively downregulated specific proteins associated with endothelial cell proliferation and migration, aortic luminal occlusion, and myocardial failure, thereby demonstrating significant cardioprotective potential.

Atherosclerosis, a major pathological driver of CVDs, is primarily triggered by low-density lipoprotein (LDL) oxidation, chronic inflammation, and intravascular lipid accumulation. Recent evidence has elucidated the molecular mechanisms through which HT mitigates atherogenesis. Notably, HT protects vascular endothelial cells against oxidative injury by activating the Nrf2/HO-1 signaling pathway through the PI3K/Akt and ERK1/2 pathways, thereby reducing intracellular reactive oxygen species (ROS), which may contribute to the prevention of atherosclerosis [50]. In addition, HT modulates autophagy in vascular adventitial fibroblasts (VAFs) via the SIRT1-mediated Akt/mTOR axis. This regulation attenuates vascular inflammation and provides additional protection against atherosclerotic plaque formation [51].

### 5.4. Antitumor Activity

HT is capable of inhibiting the proliferation of various tumor cells through mechanisms such as cell cycle arrest and apoptosis induction.

Parra-Perez [52] evaluated the anticancer activity of HT against hematological malignancies using in vitro cell models. Acute human leukemia T-cell lines (Jurkat and HL60) and murine macrophages (RAW264.7) were employed. Cytotoxicity assays yielded IC_50_ values of 27.3, 109.8, and 24.8 µg·mL^−1^ for Jurkat, HL60, and RAW264.7 cells, respectively, after 24 h of exposure. Results demonstrated that HT arrested both Jurkat and HL60 cells in the G_0_/G_1_ phase, and the S-phase cell population was significantly reduced. Combined apoptosis and cell cycle assays confirmed the antiproliferative effect of HT. The compound decreased the percentage of dividing cells and increased apoptosis rates. Mechanistically, HT inhibited the PI3K signaling pathway, which in turn activated the MAPK pathway. Changes in the expression of inflammatory and cancer-related markers further supported these findings. In LPS-stimulated RAW264.7 cells, HT reduced NO levels and modulated the expression of related markers. These findings suggest that HT holds potential as a natural therapeutic agent against hematological malignancies, including leukemias, myelomas, and lymphomas.

### 5.5. Anti-Osteoporotic Activity

HT has demonstrated significant anti-osteoporotic activity. In an in vitro study, Hioki et al. [53] investigated the effect of HT on TNF-α-induced M-CSF release in osteoblast-like MC3T3-E1 cells. Experimental results showed that HT inhibited M-CSF release, likely through attenuation of TNF-α-induced phosphorylation of Akt and p44/p42 MAP kinases, leading to the downregulation of M-CSF mRNA expression. Oleuropein, another major olive polyphenol, exerted comparable inhibitory effects in the same study. Additionally, HT significantly reduced TNF-α-induced IL-6 secretion. These effects collectively inhibit osteoclast proliferation and differentiation, thereby preventing excessive bone resorption. Therefore, this study indicates that HT exhibits favorable biological activity for the prevention of osteoporosis.

### 5.6. Hypoglycemic and Hypolipidemic Activities

The hypoglycemic efficacy of HT has also been validated in in vivo studies. Hamden et al. [54] isolated and purified HT from fresh olive mill wastewater (OMW) and evaluated its effects in alloxan-induced diabetic rats. HT administration led to a significant reduction in fasting plasma glucose levels, which was attributed to inhibition of glucose auto-oxidation and attenuation of advanced glycation end product (AGE) accumulation.

HT also exhibits lipid-lowering activity. In a mouse model of metabolic syndrome induced by a high-fat/high-sugar diet, 16 weeks of supplementation with an HT-enriched subcritical water extract of OP markedly decreased plasma triglyceride concentrations and elevated HDL-cholesterol levels [55]. The potential mechanisms underlying these effects may involve the upregulation of uncoupling protein 1 (UCP-1) in adipose tissue, which accelerates fat oxidation and suppresses lipid deposition. This process is accompanied by enhanced phosphorylation of AMP-activated protein kinase (AMPK) and protein kinase B (AKT) in skeletal muscle. Activated phosphorylated AMPK (p-AMPK) further promotes mitochondrial biogenesis, thereby enhancing lipid oxidation.

**Figure 4:**
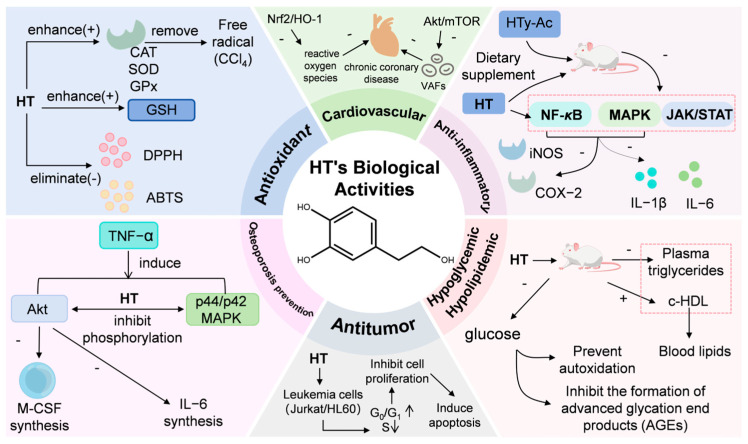
Biological activities of hydroxytyrosol in olive pomace. The central structure depicts the chemical structure of hydroxytyrosol. The diagram illustrates six major biofunctional properties of HT and its derivatives, with detailed mechanistic pathways. Arrows denote the signaling pathways, while the accompanying text labels explicitly indicate the promotion/activation or suppression/inhibition effects.

Collectively, HT exhibits a broad range of well-documented bioactivities, including antioxidant, anti-inflammatory, cardiovascular-protective, antitumor, anti-osteoporotic, and glucolipid-regulating effects (Figure 4). These properties highlight its considerable potential for future research and development.

A summary of the biological activities of HT and the concentrations tested in each assay is given in Table 6.

## 6. In Vivo Absorption and Bioavailability of HT

### 6.1. Rapid Absorption Versus Extensive First-Pass Metabolism

HT possesses physicochemical properties that favor rapid transmembrane absorption. Its low molecular weight, amphiphilic character, and high polarity allow it to passively cross lipid bilayers, including intestinal epithelial membranes and the blood–brain barrier [56]. Following oral administration, HT is rapidly and almost completely absorbed in the upper gastrointestinal (GI) tract. Vissers et al. [57] confirmed this efficient uptake in a clinical study involving healthy subjects and patients with an ileostomy. After consumption of 100 mg of olive oil phenols, negligible amounts of free HT were recovered in the ileostomy effluent, indicating that absorption occurs predominantly in the upper small intestine before reaching the colon.

Despite this highly efficient absorption, the systemic bioavailability of free HT remains low [56]. Upon absorption across the intestinal mucosa, HT undergoes extensive first-pass metabolism initiated in the intestinal mucosa and subsequently in the liver [58], where the parent compound is rapidly converted into glucuronide conjugates, sulfate conjugates, and, to a lesser extent, methylated derivatives [59]. Once absorbed, HT is rapidly metabolized in the gut and liver into glucuronide and sulfate conjugates, as well as methylated derivatives [60]. The extensive presystemic metabolism of HT results in low systemic exposure to the free parent compound, which represents a major limitation for its functional application [59].

Before absorption can occur, however, HT must first survive the harsh environment of the GI lumen, a topic addressed in the following section.

### 6.2. In Vitro Digestion Models and Bioaccessibility

Before being absorbed by the intestinal epithelium, ingested HT must survive the complex luminal environment of the gastrointestinal tract. The fraction of HT that remains stable under these conditions and is released from the food matrix is referred to as the bioaccessible fraction. Standardized in vitro digestion models, such as the INFOGEST protocol, have been developed to evaluate this process under controlled conditions [61]. Reboredo-Rodríguez et al. [62] demonstrated that during gastric digestion of EVOO, extensive hydrolysis of secoiridoids occurs, leading to the release of free HT, tyrosol, and HT acetate. Upon transition to the intestinal phase, further degradation of secoiridoids was observed, with the liberated simple phenols primarily partitioning into the aqueous phase, where they become available for absorption.

Current evidence from in vitro digestion models shows that HT stability depends on the digestive compartment. These models typically simulate the oral, gastric, and intestinal phases at 37 °C [63]. The intestinal phase uses pH 6.5 to 7.5, pancreatin, and bile salts [63]. These conditions are used to test HT stability in different delivery systems. In simple emulsions and double emulsions, gastric digestion reduces HT content by 11% and 14%, respectively. Intestinal digestion reduces HT by 10% and 7%, respectively. The total HT loss is about 21% [64]. In simple emulsion and double emulsion systems, the combined gastric and intestinal phases cause a total HT loss of about 21%. In gelled double emulsion, the loss is lower. HT decreases by about 4% in the gastric phase and 7% in the intestinal phase [64]. The gel network slows down triacylglyceride hydrolysis and delays HT exposure to digestive fluids. In addition, proteins such as gelatin release peptides and amino acids with antioxidant activity during digestion. These products may help protect HT from degradation [64].

Phenolic compounds interact with food macromolecules, including carbohydrates, proteins, and lipids, through hydrophobic forces, hydrogen bonds, and, in some cases, covalent linkages with proteins [65]. These interactions affect HT bioaccessibility in two opposing ways. Binding to the food matrix can protect HT from oxidative degradation during gastrointestinal transit. At the same time, it can restrict HT release and lower the fraction available for absorption [65]. The overall effect depends on the type of phenolic compound, the composition of the matrix, and the processing conditions applied.

Human data confirm the relevance of the food matrix for HT absorption. Alemán-Jiménez et al. [66] administered 5 mg of HT to volunteers in different vehicles, including extra virgin olive oil, refined olive oil, flax oil, grapeseed oil, margarine, and pineapple juice. Plasma HT concentrations after 30 min were highest with extra virgin olive oil (3.79 ng/mL). Margarine and pineapple juice produced no significant increase [66]. Urinary HT recovery followed the same trend. Extra virgin olive oil yielded 0.86 μg/mg creatinine, compared with 0.63, 0.55, and 0.33 μg/mg creatinine for refined olive, flax, and grapeseed oils, respectively [66]. These findings show that the lipid component of the matrix, and particularly the composition of extra virgin olive oil, favors HT solubilization and uptake. The food matrix ultimately determines the fraction of HT that remains available for absorption.

### 6.3. Interactions with the Intestinal Microbiota

A substantial fraction of dietary HT precursors escapes absorption in the upper gastrointestinal tract and reaches the large intestine. Oleuropein, a major secoiridoid in olive oil, is not absorbed in the small intestine in its parent form [67]. Under acidic conditions in the stomach, conjugated forms of olive oil polyphenols undergo rapid non-enzymatic hydrolysis. Corona et al. [67] observed that after 30 min of incubation under simulated gastric conditions, the amounts of free HT and tyrosol increased 4.75- and 3.25-fold, respectively. This gastric biotransformation effectively raises the quantity of free HT entering the small intestine for absorption. Despite this, a significant portion of oleuropein survives gastric and intestinal transit and reaches the large intestine, where it is rapidly degraded by the colonic microbiota. Fermentation of oleuropein by human fecal inocula yields HT as the main metabolite, with hydroxytyrosol acetate, elenolic acid, and oleuropein aglycone also detected as intermediates [68]. Oleuropein was deglycosylated to oleuropein aglycone, which was then hydrolyzed to elenolic acid and HT, probably through microbial esterase activity [68]. Mosele et al. [68] further reported that HT itself undergoes partial degradation during the first six hours of fermentation and then remains relatively stable for up to 48 h. The main end products of this microbial catabolism were phenylacetic acid.

While the microbiota transforms HT and its precursors, HT and related olive polyphenols also influence the composition of the microbial community. Conterno et al. [69] investigated the effect of OP-enriched biscuits delivering 17.1 mg/100 g of HT and its derivatives in mildly hypercholesterolemic subjects. After eight weeks of consumption, significant increases in the urinary excretion of microbially derived phenolic acids were observed, including homovanillic acid and 3,4-dihydroxyphenyl acetic acid [69]. These small phenolic acids derive from the combined activities of host phase I and II biotransformation and the action of the gut microbiota [69]. Modest changes in the relative abundance of certain bacterial genera were also detected, with a small increase in bifidobacteria observed across multiple analytical methods [69]. Martín-Peláez et al. [70] reported that ingestion of a virgin olive oil enriched with a combination of olive and thyme phenolic compounds led to an increase in Bifidobacterium numbers, suggesting a potential prebiotic effect of these phenolics when administered together with complementary phenolic sources.

Host factors add another layer of complexity. Conterno et al. [69] observed sex-related differences in the urinary excretion of phenolic metabolites after the dietary intervention. Men excreted significantly more 3,5-dihydroxybenzoic acid, 4-hydroxyphenylacetic acid, and naringenin than women. The relative abundance of several bacterial genera, including Akkermansia, Bifidobacterium, and Bacteroides, also differed between the sexes in fecal samples collected after the ingestion of OP biscuits [69]. Domínguez-Perles et al. [71] similarly reported that female rats excreted higher amounts of HT derivatives than males after oral administration of equivalent doses of HT, hydroxytyrosol acetate, or DOPAC. These findings indicate that biological sex influences the metabolic fate of HT and its interaction with the gut microbiota.

The phenolic acids generated through microbial fermentation can be absorbed and enter the systemic circulation [69]. In the in vivo study by Mosele et al. [68], a significant increase in free HT was detected in fecal samples after three weeks of daily intake of phenol-rich olive oil, confirming that a portion of dietary HT reaches the large intestine and undergoes microbial transformation. These challenges underscore the need for encapsulation and controlled release strategies that protect HT from premature degradation and extend its bioavailability.

### 6.4. Encapsulation and Controlled Release Strategies

The clinical use of HT in therapeutic formulations and functional foods is constrained by its physicochemical properties [59]. Structurally, HT is a derivative of catechol. The phenolic OH group provides antioxidant potential through H^+^ and electron donation [72]. However, this catechol configuration oxidizes easily [73], and the hydroxyl groups are highly sensitive to light and oxygen [59]. As a result, HT oxidizes readily during storage and preparation [59,73]. In addition, HT is a polar molecule with low solubility in oil. This restricts its direct use in fat-rich matrices [59]. During gastrointestinal transit, HT also degrades under the alkaline conditions of the intestinal phase [63,64]. Encapsulation and controlled release strategies have been developed to overcome these limitations (Figure 4).

Emulsion-based systems can encapsulate and protect HT during storage and digestion. In water-in-oil emulsions, HT is dissolved in aqueous droplets dispersed within a continuous oil phase [74]. Lipophilic emulsifiers stabilize the droplet interface and shield HT from direct exposure to degradative conditions [64,74]. More complex structures offer additional control over HT release. Double emulsions contain oil globules that enclose water droplets, all dispersed in an external aqueous phase [64]. Gelled double emulsions further immobilize these structures within a hydrogel network, offering additional protection and slower release [75]. Microemulsions, which form spontaneously and remain thermodynamically stable, provide better long-term storage stability than conventional emulsions, although conventional emulsions allow faster HT release [64].

Liposomes have also been tested as HT carriers. These nanoscale vesicles are composed of one or more phospholipid bilayers surrounding an internal aqueous core [59]. Their amphiphilic structure allows them to carry both hydrophilic and lipophilic compounds [59]. Baréa et al. [76] used synthetic liposomes that mimicked the lipid membrane composition of olive extracellular vesicles. The liposomes were loaded with HT, hydroxytyrosyl dodecanoate, rosmarinic acid, or eicosyl rosmarinate. The loaded synthetic liposomes boosted the antioxidant effect of these compounds. This effect was strongest for the lipophilic esters [76]. The vesicular structure limits the contact of HT with external oxidizing agents and helps maintain its functional activity.

Structural encapsulation traps HT inside a protective polymeric structure [77]. These systems protect HT from degradation caused by light, oxygen, pH fluctuations, and enzymatic reactions [59]. They also allow controlled release of the encapsulated compound [59]. Capsules consist of a hollow cavity enclosed by a polymeric shell. Spheres embed HT within a solid polymer matrix [77]. The encapsulating material can be chosen from natural polymers, such as chitosan, gelatin, or albumin, or from synthetic ones, such as poly lactide co-glycoside acid and poly-lactide acid [77].

**Figure 5:**
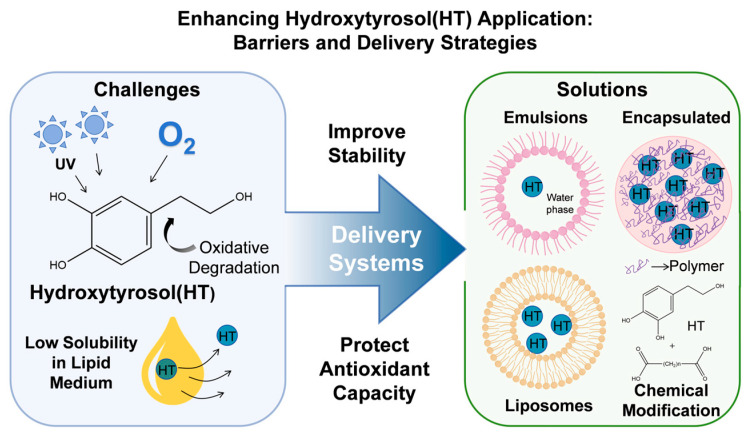
Barriers to hydroxytyrosol (HT) application and corresponding delivery strategies. HT is susceptible to oxidative degradation and exhibits low lipid solubility (left), which can be addressed using emulsion, liposome, encapsulation, and chemical modification systems (right).

Chemical modification alters the properties of HT itself rather than placing it inside a carrier. Lopes et al. [78] synthesized HT bis-ester derivatives, each containing two polyphenolic moieties linked by an alkyl spacer of different length. The C12 derivative was the most effective antioxidant in soybean phospholipid liposomes. It increased oxidative stability by 2.2 times relative to the control [78]. When the derivatives were added during liposome preparation instead of after liposome preparation, the antioxidant capacity rose further to about 2.8 times above the control [78]. The authors attributed this improvement to a probable transmembrane arrangement in which both polyphenolic rings were located near the phospholipid polar heads [78]. The major barriers to HT application, together with the four core delivery strategies discussed above, are summarized in Figure 5.

Beyond the formulation approaches described above, nanoscale delivery systems have also been explored to improve the oral bioavailability of HT. Lipid-based nanoparticles are among the most studied carriers for this purpose [79]. Solid lipid nanoparticles (SLNs) and nanostructured lipid carriers (NLCs) use solid lipid matrices to encapsulate drugs and can load both hydrophilic and hydrophobic molecules [79]. These lipid carriers are made from naturally occurring lipids that are digested by the body, which reduces concerns about formulation toxicity [79]. Chitosan nanoparticles represent a polymeric alternative. HT has been loaded into such particles and then embedded within a thermosensitive hydrogel composed of Pluronic F-127 and hyaluronic acid [80]. This combined system is fluid at room temperature, allowing injection in a minimally invasive manner, and transitions to a gel at body temperature. Once formed, the gel sustains HT release at the target site [80].

Polymer-coated liposomes have also been developed for oral HT delivery. De Leo et al. [81] prepared liposomes co-loaded with HT and curcumin, two antioxidants that differ in polarity. The liposomes were shielded by two concentric polymeric layers. The inner coating consisted of polyethylene glycol, and the outer shell was made of Eudragit S100, a gastro-resistant polymer [81]. During in vitro digestion, the outer Eudragit layer protected the liposomes from mouth and gastric fluids and dissolved only upon reaching the intestinal environment. The inner polyethylene glycol layer then provided resistance against bile salts and pancreatic enzymes [81]. This dual-coating strategy helped intact liposomes reach distal intestinal regions, where near-neutral pH and lower enzymatic activity favor HT absorption [81].

A shared feature of these nanoscale systems is their capacity to protect encapsulated compounds from degradation in the gastrointestinal environment, enhance drug solubility and permeability, and improve oral bioavailability [82]. Lipid carriers can also traverse gastrointestinal barriers and access the lymphatic pathway, thereby bypassing hepatic first-pass metabolism [83]. Coating lipid nanoparticles with muco-adhesive polymers may further improve absorption by prolonging contact with the intestinal epithelium [83]. Despite these advances, the application of nanoscale delivery systems to HT remains at an early stage. Most evidence derives from in vitro assays and proof-of-concept studies. Moving toward clinical and commercial use will require addressing formulation stability at scale, long-term safety, and in vivo efficacy.

Collectively, the delivery strategies reviewed in this section address the principal barriers that limit HT bioavailability. Emulsions protect HT during gastrointestinal transit and enable controlled release. Liposomes and polymer-coated vesicles offer versatility in loading both hydrophilic and lipophilic forms and can be engineered for site-specific delivery. Structural encapsulation within polymeric matrices provides a physical barrier against oxidation. Chemical modification tunes the physicochemical properties of HT for improved compatibility with lipid-based carriers. Nanoscale systems further extend these capabilities by enhancing solubility and permeability. However, the majority of these approaches have been evaluated only in vitro. Their performance under in vivo conditions, their long-term safety, and their feasibility for large-scale production remain open questions for future research.

## 7. Industrial Translation, Sustainability, and Future Perspectives

### 7.1. Technology Readiness and Scale-Up Considerations

The industrial feasibility of HT recovery depends on the TRL of each unit operation. Table 2 and Table 4 summarize the estimated TRL and scalability of the extraction and purification methods reviewed in Section 4.1 and Section 4.2. Among extraction methods, solvent extraction and UAE hold the highest TRL (7–8). UAE has been demonstrated at the pilot scale by Rodríguez et al. [84], who achieved a polyphenol yield of 3.0 g GAE/L at the 120 kg scale, which was 58% higher than that achieved by conventional extraction without ultrasound assistance. MAE (TRL 4–5) and EAE (TRL 5–6) occupy intermediate positions, constrained by microwave penetration depth and enzyme cost, respectively [32,33]. SFE and PLE (TRL 4–5) produce high-purity extracts but require expensive high-pressure equipment [34,35]. NADES extraction remains at the lowest readiness level (TRL 3–4), with no pilot-scale studies reported and solvent recovery unresolved [37,38].

On the purification side, membrane separation and LLE are the most mature technologies (TRL 7–8). Romeu et al. [40] validated membrane technology in an industrial setting, processing up to 1500 kg of pomace per week. SPE (TRL 4–5) achieves high HT purity at the laboratory scale but has not been demonstrated at the pilot scale [41]. Crystallization and derivatization (TRL 3–4) improve HT stability but lack pilot-scale validation and food safety evaluation [43,44].

A critical obstacle to industrial implementation is the absence of integrated purification trains connecting extraction, pre-concentration, enrichment, and final purification. The variability of the starting biomass, discussed in Section 3.2, further complicates process standardization.

Limited techno-economic analyses support the feasibility of HT recovery at scale. Orive et al. [85] proposed a biorefinery for EOP combining polyphenol recovery with anaerobic digestion of the dephenolized fraction. For a plant treating 1500 t of EOP per year, the NPV was €1,996,856, with the minimum sales volume and selling price of the hydroxytyrosol extract identified as the most influential factors for profitability. A second study treating 8000 t of two-phase pomace per year reported an NPV of €1,493,234 and an IRR of 12.8% [86]. Pérez-Almada et al. [87] designed a multiproduct biorefinery producing antioxidant extracts, lignin, and bioethanol from EOP. The base scenario showed a cost of 10.99 USD per functional unit, while the integration of carbon capture and storage (CCS) achieved a negative carbon footprint (−1.05 kg CO_2_-eq per functional unit) at the expense of a 2.5-fold cost increase [87].

In summary, solvent extraction, UAE, membrane separation, and LLE are best positioned for near-term industrial implementation. Emerging approaches such as NADES, cocrystallization, and derivatization require advances in scalability before industrial use can be considered. Integrated pilot-scale demonstrations and comprehensive techno-economic assessments remain priorities for advancing HT recovery toward commercial application.

### 7.2. Sustainability and Life Cycle Assessment

The environmental impacts of olive oil production have been studied through LCA for over a decade. Espadas-Aldana et al. [88] reviewed 23 LCA studies and found an average carbon footprint of about 460 kg CO_2_-eq per ton of olives and 1.6 kg CO_2_-eq per liter of olive oil. The farming phase is the main source of environmental impact. Fertilization, pesticide use, and irrigation account for the largest share [88]. Fernández-Lobato et al. [89] studied virgin olive oil production in the Andalusian region of Spain. They reported a carbon footprint between 1.93 and 3.00 kg CO_2_-eq per kg of olive oil. Pomace valorization contributed around 18.6% of the total [89].

No complete LCA has been published for HT recovery from OP. This gap is significant. Most claims about the sustainability of different extraction and purification methods are qualitative. They rely on solvent type and energy use rather than on measured environmental data. Evidence from other agricultural residues shows why HT-specific assessments are needed. Arias et al. [90] evaluated the LCA of phenolic compound recovery from orange peel and tomato seed. They compared solvent extraction, ultrasound-assisted extraction, microwave-assisted extraction, and subcritical water extraction. Energy use was the main driver of environmental impacts across all technologies [90]. Another study focused on antioxidant recovery from wine residues. The authors compared subcritical water extraction, ultrasound-assisted extraction, solvent extraction, and microwave-assisted extraction. Microwave extraction caused the highest environmental impact because of low yields and high energy demand during freeze drying. Subcritical water extraction performed best, partly because it used only water as the solvent [91]. A direct comparison of UAE and MAE for polyphenol extraction from beet seeds reached a similar conclusion. The authors combined LCA with productivity and energy-use metrics in a multi-criteria optimization. MAE gave higher polyphenol concentrations and antioxidant activity. UAE cut energy use by up to 3.6 times and reduced climate change impacts by up to 12% [92]. These studies show that the choice of extraction method, solvent, and feedstock all affect the environmental outcome. This further underlines the need for HT-specific LCA data.

Several LCA studies on OP management offer useful benchmarks. Benavente et al. [93] compared hydrothermal carbonization, composting, anaerobic digestion, and incineration with energy recovery. Fernández-Lobato et al. [94] studied an integrated gasification plant for OP placed directly at the oil mill. The gasification scenario cut the carbon footprint of olive oil production from 2.21 to 1.74 kg CO_2_-eq per kg. It also turned the industrial phase into a carbon sink [94]. Pérez-Almada et al. [87] designed a multiproduct biorefinery that produces antioxidant extracts, lignin, and bioethanol from exhausted OP. The base scenario cost 10.99 USD per functional unit. Adding carbon capture and storage brought the carbon footprint below zero, reaching −1.05 kg CO_2_-eq per functional unit, but raised the cost by a factor of 2.5 [87]. This study assessed the whole biorefinery, not HT recovery alone. It did not compare different extraction or purification methods.

Future LCA work on HT recovery should address several points. The environmental burden of solvent production and recovery needs to be quantified, since water, ethanol, ethyl acetate, and NADES components differ widely in their impacts. Drying steps deserve special attention. In polyphenol extraction LCAs, drying has been identified as a major source of greenhouse gas emissions [91]. Energy use across different extraction methods must be measured, as the comparison between UAE and MAE has shown that technology choice can change the environmental profile considerably [92]. Waste streams should be included within the system boundaries. Spent resins, organic effluents, and the solid residue left after extraction all carry an environmental cost. Finally, HT recovery needs to be compared with current pomace disposal practices, such as direct combustion or composting. Without this comparison, it is impossible to say whether HT recovery brings a real environmental benefit.

### 7.3. Commercial Products and Regulatory Aspects

HT has moved from the laboratory research stage to the commercial market, where it is most commonly found in the form of dietary supplements. Lopez-Huertas and Fonolla administered 45 mg of purified HT (99.5% purity) daily for eight weeks to volunteers with mild hyperlipidemia and found that the dose was safe and did not influence markers of cardiovascular disease, blood lipids, inflammatory markers, or liver or kidney function [95]. Notably, serum vitamin C levels increased by twofold at weeks four and eight compared with baseline, indicating a physiologically relevant antioxidant function [95].

HT has also been incorporated into a range of food matrices. In refined oils, polyphenol extracts enriched with HT preserved α-tocopherol content more effectively than butylated hydroxytoluene, and similar extracts enhanced the oxidative stability of sunflower oil, primarily due to the presence of HT and oleuropein [96,97]. In wines, HT replaced sulfur dioxide, intensifying color, scents, and taste at bottling while providing antioxidant effects during storage [98,99]. Beer supplemented with olive extract showed increased antioxidant activity and color, although concentrations above 1% impaired foam stability [100]. The addition of OP to coffee increased phenolic content and antioxidant capacity, with a 10% pomace blend achieving the highest bioactivity and 30.5% α-amylase inhibition at 200 μg/mL [101]. In dairy products, HT addition to yogurts preserved organoleptic properties, improved antioxidant activity and lactic acid bacteria growth, and contributed to reducing LDL cholesterol, body weight, and blood pressure [102]. In meat and fishery products, HT decreased lipid and protein oxidation in sausages and patties while preserving color, flavor, and texture and extending shelf life through antimicrobial effects [103,104]. In bakery products, biscuits fortified with 5.25 mg of HT per 30 g serving darkened in color and reduced oxidized LDL levels in volunteers [105].

Regarding regulatory status, HT is the only phenolic compound that has received a European Food Safety Authority health claim approval [95]. EFSA has also authorized chemically synthesized HT as a novel food, specifying permitted food categories, including fish and vegetable oils up to 215 mg/kg and margarines up to 175 mg/kg [106]. The safety assessment established a no-observed-adverse-effect level of 50 mg/kg body weight per day, and the Panel concluded that the margins of exposure were sufficient for the target population. The authorization excludes children under 36 months of age, pregnant women, and breastfeeding women [106].

### 7.4. Future Directions

Despite significant advances in the recovery of HT from OP, several critical gaps persist that hinder the transition from laboratory research to industrial implementation.

The intrinsic compositional variability of OP remains a fundamental challenge for industrial reproducibility. Agronomic factors, maturity index, and extraction systems create divergent phenolic profiles in the resulting pomace [20,21,22,23,24]. Currently, no standardized protocols exist for rapid, non-destructive characterization of incoming pomace batches. Future research should develop spectroscopy-based grading systems to classify pomace according to its HT precursor content, enabling dynamic adjustment of extraction parameters and improved batch-to-batch consistency.

Green extraction technologies, particularly natural deep eutectic solvents (NADES), have demonstrated remarkable potential [107,108,109]. However, their industrial translation is constrained by two unresolved barriers. First, the high viscosity and non-volatility of NADES create a major bottleneck for product recovery [110]. Second, the food-grade status of most NADES formulations remains unevaluated [111]. Future research should clarify the safety profiles of these solvents and develop more stable and efficient NADES systems. Computational tools such as COSMO-RS offer promising avenues for NADES design [112], but experimental validation across diverse olive cultivars is still required.

Downstream purification represents another area where integrated approaches are urgently needed. While membrane separation and resin adsorption have each been demonstrated individually [39,40], a fully integrated, sequential purification train specifically optimized for HT from OP remains unrealized. The combination of ultrafiltration and nanofiltration, selective resin adsorption, and other separation techniques has shown promise [113,114]. However, these unit operations have not been coupled and validated at the pilot scale. The long-term stability of resins over repeated adsorption–desorption cycles and the techno-economic feasibility of integrated purification trains remain to be quantified.

The biological performance of recovered HT is intimately linked to its bioavailability, yet current evidence reveals substantial gaps between in vitro models and in vivo reality. In vitro studies indicate that the food matrix and gender differences significantly influence HT metabolism and gut microbiota interactions [66,115]. Although encapsulation strategies have shown promise in protecting HT from gastrointestinal degradation [59,81], the majority have been evaluated only in vitro or in animal models. Long-term safety data for nanoscale delivery systems are absent, and the influence of individual gut microbiota composition on formulation efficacy has not been systematically addressed. Furthermore, whether the high bioaccessibility observed for NADES extracts during simulated digestion translates into enhanced systemic exposure in humans remains unknown [108]. It is therefore essential to conduct clinical pharmacokinetic studies comparing free HT derived from OP with encapsulated or NADES-formulated HT.

Finally, the environmental and economic sustainability of HT recovery processes requires rigorous quantification. Although qualitative assessments suggest favorable profiles for aqueous extraction and membrane separation [28,40], comprehensive life cycle assessments comparing different extraction and purification routes are currently lacking [88,89,90]. Integrated biorefinery concepts that simultaneously recover multiple products from the same pomace batch have been proposed [41], but techno-economic analyses validating the profitability of such multiproduct schemes remain scarce [85,86]. Future research must incorporate comprehensive life cycle assessment and techno-economic analysis into process design to ensure that laboratory-optimized methods genuinely reduce environmental burdens.

## 8. Conclusions

This review surveyed the occurrence of HT in OP, the variables that determine its formation, and the current methods available for its extraction, purification, and detection. The main biological activities reported for HT and its in vivo absorption behavior were also examined. Nevertheless, moving from laboratory findings to industrial application remains difficult. The composition of OP varies widely with cultivar, maturity, irrigation history, and milling technology, which makes it difficult to achieve consistent process performance. A fully integrated purification train, from membrane pre-concentration through resin enrichment to final crystallization, has not yet been tested at the pilot scale. Life cycle assessments that compare alternative extraction and purification routes are lacking. Most delivery systems developed to protect HT and improve its bioavailability have only been evaluated in vitro, and their in vivo behavior remains unverified. Future work needs to focus on constructing and pilot-testing integrated purification schemes, generating life cycle inventory data for HT recovery processes, and conducting rigorous pharmacokinetic and long-term safety studies of encapsulated HT formulations in vivo. Addressing these gaps is necessary to turn OP from an environmental burden into a commercially viable and sustainable source of high-value bioactive compounds.

## Figures and Tables

**Table 2:** Technology readiness, scalability, sustainability, and downstream compatibility of hydroxytyrosol extraction methods.

Method	TRL	Scalability	Environmental Sustainability	Food-Grade Compatible	Impact on Downstream Purification	Ref.
SE	7–8	High	High	Yes	Directly compatible with membrane/resin separation	[28]
UAE	7–8	High	Medium	Yes	EtOH removal required before purification	[27]
UAE	7–8	Medium	High	Yes	Minimal EtOH removal; LC-ready	[31]
MAE	4–5	Medium	High	Yes	Directly compatible with membrane/resin	[32]
EAE	5–6	Medium	High	Yes	Directly compatible with membrane/resin	[27]
EAE	5–6	Medium	High	Yes	Directly compatible with membrane/resin	[33]
SFE	4–5	Low–Medium	Medium	Yes	CO_2_ self-separates; a clean extract aids purification	[34]
PLE after scCO_2_ defatting	4–5	Medium	Medium–High	Yes	EtOH removal and freeze drying required	[35]
NADES extraction	3–4	Very low	Medium	Pending	Major bottleneck: product recovery from DES	[37]
NADES extraction	3–4	Very low	Medium	Pending	Major bottleneck: product recovery from DES	[38]

Note: TRL (Technology Readiness Level) was conservatively estimated based on the experimental scale reported in each cited study. Scalability was assessed by considering equipment availability and the existence of pilot- or industrial-scale demonstrations. Environmental sustainability was qualitatively evaluated based on solvent type, energy demand, and waste generation. Food-grade compatibility refers to whether the solvent and processing aids are approved for food applications. A complete LCA study for HT extraction methods is currently lacking. Abbreviations: DES, deep eutectic solvent; EAE, enzyme-assisted extraction; EtOH, ethanol; HT, hydroxytyrosol; LCA, life cycle assessment; LC, liquid chromatography; MAE, microwave-assisted extraction; NADES, natural deep eutectic solvent; PLE, pressurized liquid extraction; scCO_2_, supercritical carbon dioxide; SFE, supercritical fluid extraction; TRL, technology readiness level; UAE, ultrasound-assisted extraction; Ref., Reference.

**Table 3:** Comparison of purification methods for hydroxytyrosol from olive pomace.

Purification Method	Key Solvents/Materials	Recovery/Enrichment	Final Purity	Processing Conditions	Main Advantages	Main Limitations	Ref.
Membrane separation	Water; NF270; RO	7–9× HT enrichment; 100% TPC rejection (BW30)	HT: ~15% in dry residue; TPC: 1234.3 mg GAE/L	NF: pilot-scale, 10 bar; RO: ~50 bar; 1500 kg pomace → 80 L concentrate/week	Industrially validated; no organic solvents; product stable ≥14 months	HT yield depends on free HT in feed; membrane fouling; regular cleaning required	[40]
Liquid–liquid extraction	Ethyl acetate; 1:2 (*v*/*v*) extract-to-solvent, two steps	HT: 88.8%	HT: 118 mg/g; TPC: 273 mg/g	15 min stirring, 1250 rpm, room temperature	Highest HT recovery among the methods evaluated by [41]; simple equipment; high selectivity for HT over glucose and mannitol	Organic solvent required; lower TPC recovery than SPE	[41]
MAE-DLLME	1-Octanol; ethanol; water: ethanol (40:60)	HT: 98.7%	Not reported	MAE: 220 W, 12 min; DLLME: 2 min shaking + 10 min centrifugation	High recovery; fast; low solvent consumption; low LOD	Analytical-scale only; 1-octanol not food-grade	[42]
SPE (DSC-8)	DSC-8; ethyl acetate	HT: 30.85% (ethyl acetate); 31.5% (total)	HT: 291 mg/g; TPC: 873 mg/g	1 g cartridge, 5.5 mL extract, single elution	Highest HT purity among the methods evaluated by [41]; selective for HT over glucose and mannitol	Resin regeneration required; organic solvents; batch operation	[41]
Crystallization (cocrystals)	Betaine, nicotinamide, isonicotinamide	Not applicable (solid-state modification)	Cocrystal powder; purity not expressed as mg/g	Crystallization from solution at −20 °C, 24 h (HT-BET, HT-NIC, HT-INA Form I); liquid-assisted grinding (HT-INA Form II)	Solid product; high melting point; excellent stability	Requires pure HT feed; food safety not evaluated	[43]
Derivatization (acetylation)	HClO_4_-SiO_2_ + Ac_2_O; MPLC	35.6% (from OMWW extract)	Not quantified (purity confirmed by HPLC and NMR)	Reaction: 5 min, RT; MPLC: single step	Chemically stable; regenerates HT in cells; mild reaction conditions	Multi-step; safety at pharmacological doses is not established	[44]

Note: Recovery values refer to the percentage of HT recovered relative to the initial HT content in the feed extract. Final purity values are reported as given in the original studies (mg/g extract or % in dry residue). “Not reported” indicates that the corresponding data were not provided in the cited reference. “Not applicable” indicates that the corresponding concept does not apply to this method. “Not quantified” indicates that purity was confirmed qualitatively (e.g., by HPLC or NMR) but was not expressed as a numerical value in the original study. Abbreviations: Ac_2_O, acetic anhydride; BET, betaine; BW30, brackish water reverse osmosis membrane; DLLME, dispersive liquid–liquid microextraction; DSC-8, silica-based octyl (C8) adsorbent; GAE, gallic acid equivalents; HClO_4_-SiO_2_, perchloric acid adsorbed on silica gel; HT, hydroxytyrosol; INA, isonicotinamide; LLE, liquid–liquid extraction; LOD, limit of detection; MAE, microwave-assisted extraction; MPLC, medium-pressure liquid chromatography; NF, nanofiltration; NIC, nicotinamide; OMWW, olive mill wastewater; RO, reverse osmosis; RPM, revolutions per minute; RT, room temperature; SPE, solid-phase extraction; TPC, total phenolic content; *v*/*v*, volume per volume.

**Table 4:** Technology readiness, scalability, sustainability, and food-grade compatibility of hydroxytyrosol purification methods.

Method	TRL	Scalability	Environmental Sustainability	Food-Grade Capability	Ref.
Membrane separation	7–8	High	High (water-based; no organic solvents)	Yes	[40]
Liquid–liquid extraction	7–8	High	Medium (ethyl acetate requires recovery)	Requires solvent removal	[41]
MAE-DLLME	4–5	Low–Medium	Medium (1-octanol; microwave energy)	No (1-octanol is not food-grade)	[42]
SPE (DSC-8)	4–5	Medium	Medium (organic eluents; resin regeneration)	Requires solvent removal	[41]
Crystallization (cocrystals)	3–4	Low	High (no solvents during crystallization)	Pending (coformer safety not evaluated)	[43]
Derivatization (acetylation)	3–4	Low	Medium (chemical reagents; MPLC solvents)	No (stabilization strategy; not a food-grade product)	[44]

Note: TRL (Technology Readiness Level) was estimated by the authors based on the experimental scale reported in each cited study and the general maturity of the corresponding technology in downstream processing. Scalability was assessed by considering equipment availability, operational complexity, and whether pilot- or industrial-scale demonstrations have been reported. Environmental sustainability was qualitatively evaluated according to solvent toxicity, energy demand, and waste generation described in each study. These assessments reflect the judgment of the authors and are not explicitly stated in the original references. Food-grade capability indicates whether the method and its auxiliaries can be directly applied in food production without further solvent removal or regulatory approval. A complete life cycle assessment comparing the environmental impact of different HT purification methods is currently lacking. Abbreviations: DLLME, dispersive liquid–liquid microextraction; DSC-8, silica-based octyl (C8) adsorbent; HT, hydroxytyrosol; LLE, liquid–liquid extraction; MAE, microwave-assisted extraction; NF, nanofiltration; RO, reverse osmosis; SPE, solid-phase extraction; TRL, technology readiness level.

**Table 5:** Characteristic UV absorption and mass spectrometric parameters for HT in olive pomace extracts.

Parameter	Value	Ref.
Molecular formula	C_8_H_10_O_3_	[35]
UV λmax (nm)	280	[45]
ESI^−^ precursor ion [M-H]^−^ (*m*/*z*)	153	[8,35]
MS/MS product ion (*m*/*z*)	123	[8,35]
Mass difference (Da)	30	[8,35]
MRM transition	153 → 123	[8]
Fragmentor voltage (V)	100	[8]
Collision energy (eV)	10	[8]

Notes: UV λmax, maximum absorption wavelength; ESI^−^, negative electrospray ionization; [M-H]^−^, deprotonated molecular ion; MS/MS, tandem mass spectrometry; MRM, multiple reaction monitoring. The fragment ion at *m*/*z* 123 corresponds to a mass loss of 30 Da from the precursor ion (*m*/*z* 153).

**Table 6:** Biological activities of hydroxytyrosol and the tested concentrations/doses in each experimental model.

Biological Activity	Experimental Model/Assay	Concentration or Dose Tested	Ref.
Antioxidant	DPPH radical scavenging assay	AE-EOP IC_50_ = 0.71 mg/mL; AAE-EOP IC_50_ = 0.78 mg/mL	[28]
	ABTS radical scavenging assay	AE-EOP IC_50_ = 0.69 mg/mL; AAE-EOP IC_50_ = 0.72 mg/mL	[28]
	Intracellular GSH content in HepG2 cells treated with tBOOH	OPE (native) 0.8 mg/mL; OPE (hpbCD) 2.5 mg/mL; HTS 20 µM; mixture (HTS 3.7 µM plus TS 1.2 µM)	[46]
	CCl_4_-induced liver injury in rats	50 mg extract/kg body weight/day, oral gavage, 8 weeks	[28]
Anti-inflammatory	LPS-stimulated BV2 microglial cells	OPJ at 6.25, 12.5, 25, and 50 µg/mL (HT content approximately 10.92% *w*/*w*); LPS 100 ng/mL	[47]
	Pristane-induced systemic lupus erythematosus in mice	HT or HT-Ac 100 mg/kg diet (equivalent to approximately 20 mg/kg body weight/day), 6 months	[48]
Cardiovascular-protective	Proteomic analysis of aortic and cardiac tissues in healthy female Wistar rats	HT or SEC 5 mg/kg rat weight/day, oral, 21 days	[49]
	H_2_O_2_-induced oxidative injury in porcine pulmonary artery endothelial cells	HT at 10 to 100 µM (proliferation and cytoprotection); 50 µM for mechanistic studies (Nrf2/HO-1 pathway); H_2_O_2_ 500 µM	[50]
	TNF-α-stimulated rat vascular adventitial fibroblasts (autophagy and SIRT1 pathway)	HT at 25, 50, and 100 µmol/L; siRNA and inhibitor experiments at 50 µmol/L; TNF-α 5 ng/mL	[51]
Antitumor	Cytotoxicity assay in Jurkat, HL60, and Raw264.7 cells (24 h)	Jurkat IC_50_ = 27.3 µg/mL; HL60 IC_50_ = 109.8 µg/mL; Raw264.7 IC_50_ = 24.8 µg/mL	[52]
	Cell cycle arrest and apoptosis in Jurkat and HL60 cells	Jurkat: IC_50_ (24 h) approximately 27.3 µg/mL; HL60: IC_50_ (72 h) approximately 23.3 µg/mL	[52]
	Nitric oxide production in LPS-stimulated Raw264.7 macrophages	HT at 6.25, 12.5, 25, and 50 µg/mL; significant reduction observed from 12.5 µg/mL; LPS 0.1 µg/mL	[52]
Anti-osteoporotic	TNF-α-induced M-CSF and IL-6 release in MC3T3-E1 osteoblast-like cells	HT at 100, 300, and 500 µM (M-CSF decreased at 100 µM and above; IL-6 decreased at 500 µM); TNF-α 30 ng/mL	[53]
	Phosphorylation of Akt and p44/p42 MAPK in MC3T3-E1 osteoblast-like cells	HT at 500 µM, 60 min pretreatment; TNF-α 30 ng/mL, 10 min stimulation	[53]
Hypoglycemic	Alloxan-induced diabetes in male Wistar rats	Purified HT 20 mg/kg body weight/day, intraperitoneal injection, 2 months	[54]
	High-fat/high-sugar diet-induced metabolic syndrome in C57BL/6N mice	SWE COP 3% (*w*/*w*) in diet (equivalent to approximately 30 g/kg diet), 16 weeks	[55]

Abbreviations: AE-EOP, aqueous extract of exhausted olive pomace; AAE-EOP, aqueous-acetonic extract of exhausted olive pomace; OPE, olive pomace extract; hpbCD, hydroxypropyl-β-cyclodextrin; HTS, hydroxytyrosol; TS, tyrosol; OPJ, olive pomace juice; HT, hydroxytyrosol; HT-Ac, hydroxytyrosyl acetate; SEC, secoiridoids; SWE COP, subcritical water extract of California olive pomace; HFSD, high-fat and high-sugar diet; LPS, lipopolysaccharide; tBOOH, tert-butyl hydroperoxide; M-CSF, macrophage colony-stimulating factor; IL-6, interleukin-6; MAPK, mitogen-activated protein kinase; *w*/*w*, weight per weight.

## Data Availability

No new data were created or analyzed in this study. Data sharing is not applicable to this article.

## Abbreviations

The following abbreviations are used in this manuscript:

HT	Hydroxytyrosol
EOP	Exhausted olive pomace
TRL	Technological maturity
LCA	Life cycle assessment
UAE	Ultrasound-assisted extraction
SFE	Supercritical fluid extraction
MAE	Microwave-assisted extraction
MAWE	Microwave-assisted water extraction
MEAE	Microwave-enzyme-assisted extraction
DLLME	Dispersive liquid–liquid microextraction
EAE	Enzyme-assisted extraction
DES	Deep eutectic solvents extraction
LLE	Liquid–liquid extraction
HHPAE	High hydrostatic pressure-assisted extraction
HAE	Heat-assisted extraction
AAE-EOP	Ultrasound-assisted acetone extract of EOP
AE-EOP	Aqueous extract of EOP
OPJ	Olive pomace juice
DES-MA	Maltose-based deep eutectic solvent
La-Gc	Lactic acid-glucose
